# Learning Polynomial-Based Separable Convolution for 3D Point Cloud Analysis

**DOI:** 10.3390/s21124211

**Published:** 2021-06-19

**Authors:** Ruixuan Yu, Jian Sun

**Affiliations:** School of Mathematics and Statistics, Xi’an Jiaotong University, Xi’an 710049, China; yuruixuan123@stu.xjtu.edu.cn

**Keywords:** polynomial, separable, point convolution, point cloud

## Abstract

Shape classification and segmentation of point cloud data are two of the most demanding tasks in photogrammetry and remote sensing applications, which aim to recognize object categories or point labels. Point convolution is an essential operation when designing a network on point clouds for these tasks, which helps to explore 3D local points for feature learning. In this paper, we propose a novel point convolution (PSConv) using separable weights learned with polynomials for 3D point cloud analysis. Specifically, we generalize the traditional convolution defined on the regular data to a 3D point cloud by learning the point convolution kernels based on the polynomials of transformed local point coordinates. We further propose a separable assumption on the convolution kernels to reduce the parameter size and computational cost for our point convolution. Using this novel point convolution, a hierarchical network (PSNet) defined on the point cloud is proposed for 3D shape analysis tasks such as 3D shape classification and segmentation. Experiments are conducted on standard datasets, including synthetic and real scanned ones, and our PSNet achieves state-of-the-art accuracies for shape classification, as well as competitive results for shape segmentation compared with previous methods.

## 1. Introduction

**Figure 1:**
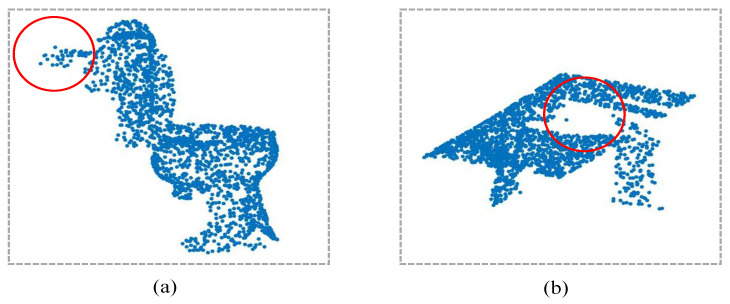
Two objects from the ScanObjectNN [8] dataset. For these objects, the points are irregular and orderless, and there are background points in subfigure (**a**) and a hole in subfigure (**b**), which are highlighted with red circles. They all bring about challenges for shape recognition.

With the development of 3D sensors, point clouds are becoming an important data type in applications such as autonomous driving, archaeology, robotics, augmented reality [1,2,3]. For these applications, shape classification and segmentation are two of the fundamental research topics, which aim to automatically recognize 3D object categories or predict point labels [4,5,6,7], and they are also the topics of our work. However, the processing of a point cloud is an intractable problem with significant challenges [4,5], i.e., the irregular and orderless properties of a point cloud make it impossible to directly apply Convolutional Neural Networks (CNNs) to them. In Figure 1, we present two objects from ScanObjectNN [8] represented by point clouds. As shown in the figure, the points are orderless, and they are irregularly distributed. Furthermore, there are noisy points from the background and holes in the point clouds. These factors all cause difficulties for the processing of a point cloud. In our work, we focus on the processing of irregular and orderless point clouds, and we aim to extract effective point features with a novel point convolution for object categorization and point cloud segmentation.

To process the irregular and orderless 3D point cloud for object category recognition or point label prediction, tremendous deep learning methods on 3D data have been proposed in recent years. Inspired by the significant success of CNNs on 2D images, some works firstly convert the point cloud to grid data and then apply CNNs to these regular data. These methods can be commonly divided into voxel-based and view-based methods. Voxel-based methods [9,10] convert 3D data to a collection of voxels and then design networks on the regular 3D voxels as in 2D images, while view-based methods [11,12] represent 3D data with images rendered from multiple views and then take the rendered images as input for their works. These methods have achieved impressive performances on various 3D tasks such as shape classification and retrieval. However, both of them need to convert raw point cloud data to voxels or images, which brings additional computational cost, and they also suffer from computational complexity brought about by 3D voxel or multi-image representations. Moreover, the voxel data usually leads to shape detail loss and data sparsity when voxelizing the point cloud. The view-based methods highly depend on camera positions to capture shape geometric details. Therefore, algorithms based on the original point cloud, i.e., point-based methods, have become a hot research field recently, directly work on the 3D objects by taking the point cloud as input. The original point cloud contains rich geometric and semantic information, so it is easier for algorithms to realize shape recognition or scene perception. Previous works have certified the advantages and successes of point-based methods for 3D shape analysis tasks such as classification, retrieval, segmentation and detection [4,5,6,7,13,14,15].

For point-based methods, to process the irregular and orderless point cloud, an essential and intractable challenge is that it is infeasible to apply standard CNNs directly to point clouds. Tremendous works have been proposed to generalize CNNs and design point convolution operations that are adaptive for point clouds. Many methods first update local pointwise features and then aggregate them by max-pooling operation to capture features with the strongest activation, without leveraging local structure [4,5,13,14]. Some works, such as [7,16,17], try to convert local point data to regular representation on-line in the network and then design traditional convolution on the converted data. The authors of [18,19] design point convolution with the help of regular representations by kernel points or weights. In addition to the strategies, many works design convolution with customizable spatial filters based on point coordinates or relations within points [6,20,21]. We present a more detailed description of point-based methods in the Related Work section. Although these point-based deep learning methods have made remarkable progress in the past years, they still face difficulty in designing an effective convolution operation for feature learning, especially for designing convolution filters that are adaptive to the irregular point clouds with noise and holes. Most of them perform better in the analysis of synthetic 3D objects, such as computer-aided design (CAD) data, which are complete, well-segmented, and noise-free, while their performances drop when operating on real scanned data [8]. We think that those drops result from the representation capability of their convolution, because the shape implied in irregular points is difficult to capture.

In our work, to deal with the irregular and orderless point clouds, we present an intuitive method to achieve a more precise approximation of ideal convolution kernels. We propose a novel point convolution, i.e., Polynomial-based Separable Convolution (PSConv), to process points, with the convolution constructed based on polynomials. This design benefits from the expressive power and approximation ability of the polynomials. Compared with previous methods, this polynomial-based strategy can better capture local shape geometry. With our PSConv as the basic layer, we further propel it with a novel and efficient strategy by a separable formulation. This separable formulation can significantly reduce the parameter size and computational cost, making it capable of building a multi-layer deep convolutional network on 3D point clouds. The primary contributions of this work are summarized as follows.

Firstly, we design a novel point convolution to extract pointwise features, with the convolution kernels constructed based on polynomials of the transformed local point coordinates. Considering that the polynomials can approximate any smooth function, our convolution kernels can approximate ideal convolution kernels and capture the local geometric information hidden behind the unstructured points.

Secondly, we propose a separable formulation for our convolution on a 3D point cloud. A simple application of our proposed point convolution would bring about huge computational cost. By this separable formation, the parameter size and computational complexity are significantly reduced. This separable convolution is efficient to apply, which makes it possible to build a deep convolutional network on 3D point clouds.

Thirdly, with our PSConv, we design a hierarchical architecture, i.e., PSNet, for 3D point cloud classification and segmentation tasks. Our PSNet achieves better or competitive performances compared with state-of-the-art methods on a standard synthetic dataset and scanned real-world dataset. For example, it achieves 93.1% OA for classification on ModelNet40 [9] and 86.2% IoU for segmentation on ShapeNet Part [22], and it also achieves the best shape classification accuracy on ScanObjectNN [8].

The rest of this paper is organized as follows. In Section 2, the literature on point-based deep learning methods is reviewed. In Section 3, we introduce the proposed method in detail. In Section 4, we evaluate our method on standard datasets, with a presentation, comparison and discussion of the results. Section 5 is the conclusion.

## 2. Related Work

### 2.1. Convolution on 3D Point Cloud

As a kind of data type, a 3D point cloud is irregular and orderless, and the traditional CNNs that work on regular data such as images can not be directly utilized. To deal with 3D object and extract pointwise descriptors directly on 3D point cloud data, various methods have been proposed.

One general strategy for point cloud analysis is to directly work on the 3D points by first updating the pointwise features and then pooling them with max-pooling operation across points. PointNet [4] pioneers these works by first designing a multi-layer perceptron (MLP) shared among points to extract a pointwise descriptor and then applying max-pooling to aggregate these point features to form a global shape descriptor, which is finally sent to Fully-Connected (FC) layers and Softmax operation for shape label prediction. PointNet++ [5] advances PointNet by applying it on local points to extract point features and then gradually coarsening the shape with the Farthest Point Sampling technique. Succeeding local PointNet and coarsening operation are applied such that a hierarchical architecture is derived. They aggregate the point features by the most coarse shape with max-pooling and finally predict the shape label with FC layers and Softmax operation. Considering that the PointNet [4] and PointNet++ [5] learn point features with MLP, more methods are proposed to propel them by various feature learning strategies. RSCNN [13] first learns to reweight point features with reweighting vectors learned by shared MLP on local geometric relations, and then the reweighted features are max-pooled and updated with another MLP. With Farthest Point Sampling, they construct a hierarchical architecture similar to PointNet++ and predict shape labels with FC layers. DGCNN [14] first computes local points with distance in feature space, within which they can calculate edges. These edges are sent to MLP to learn features at their EdgeConv layer, and the output features of the last EdgeConv layer are aggregated globally with max-pooling or average-pooling to form a global descriptor, which is used to generate classification scores. For these methods, they first update local point features and then utilize symmetric operation such as max-pooling to aggregate them, which can deal with the irregular and orderless properties of point clouds. However, max-pooling operation pools all pointwise features to be a single feature, which may ignore some detailed features encoded for the points.

Another common strategy is to design point operation by converting local irregular and orderless points to regular representation in the network, which is similar to voxel-based and view-based methods, and traditional convolution can be utilized on these regular data. PointCNN [7] first updates local point features by MLP, and then learns an X-transformer based on local point coordinates, which are utilized to reorder local point features. These reordered features are taken as regular data, on which the spatially 1D convolution can be conducted. SPLATNet [16] first interpolates input features onto a permutohedral lattice, then designs convolution over this regular lattice, whose signal is finally mapped back to points. For the work of [17], they proposed Tangent Convolution by firstly projecting local surface geometry on a tangent plane around every point. This yields a set of tangent images, and every tangent image is treated as a regular 2D grid that supports planar convolution. There are also some works that design local point convolution with the help of discrete representations, by which they can design convolution on this fixed number of discrete points or kernels. KPConv [18] defines convolution weights by kernel points, which are applied to the input points close to them, and their locations are continuous in space and can be learned by the network. InterpConv [19] utilizes discrete kernel weights and interpolates point features to neighboring kernel-weight coordinates by an interpolation function. PointGrid [23] proposes a convolutional network that incorporates a constant number of points within a grid cell. A-CNN [24] specifies the regular ring-shaped structures and directions in the computation. 3DmFV [25] utilizes the generalized Fisher Vector to achieve a fixed size representation of a possibly variable number of points in the cloud. These works try to convert local irregular points into regular formation or represent local irregular data with discrete formation, such that traditional CNNs can be employed. However, they suffer from converting raw 3D point clouds to new representations, which may be inefficient and lose geometric details of the raw point clouds.

Alternatively, some works try to generalize and learn convolution filters that are adaptive to the irregular 3D point cloud data, which are then directly utilized to conduct convolution on point clouds. These works are the most related to ours. SpiderCNN [6] first extracts k-nearest neighbors (KNN) points for every point in the shape and then designs the convolution filters as a product of a weight vector and a Taylor expansion of local point coordinates. Then, these convolution filters are employed to conduct convolution on local point features. PointWeb [20] learns convolution kernels as impact functions employed with MLP on the feature differences, which are utilized to first reweight the feature differences and then sum them up as the output of their local operator. PointConv [21] also extends traditional convolution by parameterizing a family of filters, and they treat convolution filters as non-linear functions (MLP) of the local coordinates of 3D points. These convolution filters are used for convolution on point features. The updated features are finally added up as the output of their point convolution. These works generalize traditional convolution on regular data and define point convolution for the irregular and orderless points. They focus on designing point convolution kernels based on local geometry or relations, such that they can extract point features with the help of local shape information. However, they face the challenge of designing effective and expressive kernels, which is of great importance for feature extraction. For our work, we also design point convolution kernels and aim to propose an effective solution for this challenge by learning adaptive kernels. However, we advance them and realize this idea based on polynomials of local transformed point coordinates, which benefit from the approximation and expression abilities of polynomials. Experiments also prove the efficacy of our strategy for point cloud analysis.

### 2.2. Separable Convolution

To reduce parameter size and computational cost, many works design their algorithms with the help of separable convolution [26] to construct lightweight architectures, which have been successfully applied to mobile networks [27,28]. As a special kind of spatial separable convolution, Fast Fourier Transform (FFT) rapidly converts a signal from its original domain to a representation in the frequency domain by factorizing (separating) the discrete Fourier transform matrix into a product of sparse factors. In the work of [29], they decompose the 3D filters with three 1D kernels that work in different directions separately. On the other hand, the depthwise separable convolution consists of a depthwise convolution and a pointwise convolution, and it is firstly utilized in the neural network design in the work of [30]. The depthwise convolution is a spatial convolution performed independently over each channel of an input, and the pointwise convolution is in fact a 1 × 1 convolution, which projects the output of the depthwise convolution onto a new channel space. The depthwise separable convolution is a computational effective equivalent form of the standard convolution, and it is employed as the most critical ingredient in many efficient CNN architectures such as Shufflenet [31] and MobilenetV2 [32]. Both the spatial separable convolution and depthwise separable convolution are efficient to conduct and have achieved impressive performances.

Separable convolution is also modified and utilized for 3D point cloud analysis to accelerate computational speed [7,21,33]. In the work of PointCNN [7], they adopt the depthwise separable convolution as a key step in their proposed convolution on 3D point clouds to reduce both parameter number and computational cost. Specifically, they first update the point features in feature space with MLP and then aggregate them spatially with standard 1D convolution. For PointConv [21], they reformulate their point convolution by reducing it to two standard operations, i.e., matrix multiplication and 1×1 convolution, for efficiency. For the method of SegGCN [33], the proposed fuzzy kernel is separated into the depthwise and pointwise operations to make their convolution more efficient. They firstly apply the discrete kernels to depthwise convolutions alone, following which pointwise convolution is readily achieved with 1 × 1 convolutions. For our work, we also utilize the idea of separable convolution. We do not explicitly split the convolution into depthwise and pointwise ones but advance it by separating the convolution kernels into a flexible and adaptive combination. Using this strategy, we significantly reduce the parameter size and computational cost. This efficient point convolution is also effective, as shown in the experiments.

## 3. Method

**Figure 2:**
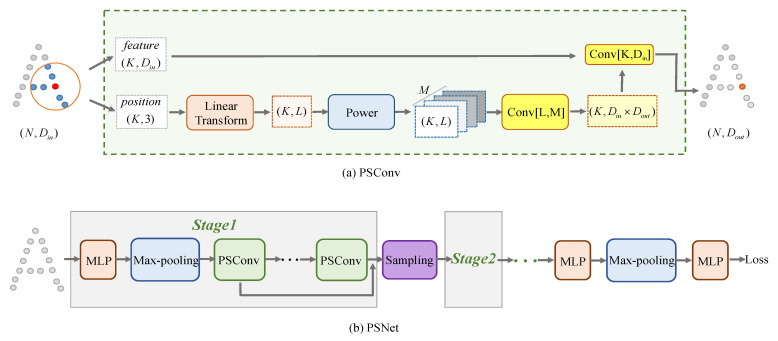
(**a**) Pipeline of PSConv. We first linearly transform local points and then compute their high-order powers to learn filters, which are utilized to conduct convolution on point features. Conv [L,M] means convolution with filter width, height as L,M. (**b**) Framework of PSNet. It is a hierarchical architecture with MLP, max-pooling, PSConv and point sampling as basic components. Please refer to Section 3 for details.

**Figure 3:**
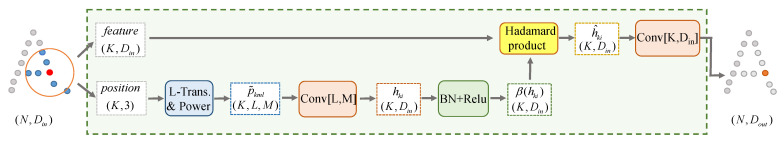
Pipeline of our separable PSConv. L-Trans. and Power denote the linear transform and power operation, which are described in Equations (1) and (2). Refer to Section 3.2 for the details of this separable PSConv.

In this section, we introduce our PSConv and PSNet in detail, with the pipeline presented in Figure 2. PSConv is our proposed convolution defined on a local point cloud, and we further propose the separable formulation of our PSConv to reduce parameter size and computational complexity, as shown in Figure 3. With our PSConv as the basis layer, we construct our PSNet with a hierarchical architecture, which can be employed for 3D point cloud classification and segmentation tasks.

### 3.1. PSConv

We aim to define an effective convolution on a 3D point cloud that directly operates on local points to extract point features. The key idea to our approach is to define a set of customizable convolution filters based on polynomials of transformed local point coordinates. Specifically, we first linearly transform the coordinates of local point cloud and then compute their high-order powers, whose polynomials are learned and utilized as convolution kernels for the convolution on point clouds. The pipeline of PSConv is shown in Figure 2a, and now we introduce it in detail.

Without loss of generality, we take local 3D points ${\left\{p_{k}\right\}}_{k=1}^{K}$$\in R^{K\times 3}$ with the corresponding feature $F={\left\{f_{ki}\right\}}_{k,i=1}^{K,D_{in}}\in R^{K\times D_{in}}$ as input for PSConv, where k,i are indices for point and feature channel, respectively. Note that ${\left\{p_{k}\right\}}_{k=1}^{K}$ denotes the centralized point coordinates by subtracting the center point coordinate of $p_{1}$, and we sort them by increasing distances to $p_{1}$. *K* is the number of local points, and we select the local k-nearest neighbors (KNN) points for center point $p_{1}$. For our PSConv, we first conduct linear transform on the coordinates of local points ${\left\{p_{k}\right\}}_{k=1}^{K}$, and achieve

(1) $$\hat{P}=\left\{{\hat{p}}_{kl}\;|\;{\hat{p}}_{kl}=\left[p_{k},1\right]\cdot {\left[a_{l},b_{l},c_{l},d_{l}\right]}^{⊤},l=1,\cdots ,L\right\},$$

where $\hat{P}\in R^{K\times L}$, [·] means the concatenation operation. ${\left\{a_{l},b_{l},c_{l},d_{l}\right\}}_{l=1}^{L}$ is the parameter to learn. To better explore the clues hidden behind $\hat{P}$ and take advantage of polynomials to approximate the ideal filters adaptively, we further introduce high-order powers of these linearly transformed points, i.e., computing *m*-order power of $\hat{P}$ as

(2) $$\tilde{P}=\left\{{\tilde{p}}_{klm}\;|\;{\tilde{p}}_{klm}={\left({\hat{p}}_{kl}\right)}^{m},m=1,\cdots ,M\right\}.$$

We take $\tilde{P}\in R^{K\times L\times M}$ as the basic element to construct our convolution filters, which are in fact a set of polynomials with learned combination parameters. Specifically, based on $\tilde{P}\in R^{K\times L\times M}$, we define the filters $G\in R^{K\times D_{in}\times D_{out}}$ of our PSConv as

(3) $$\begin{array}{c} G={\left\{g_{kij}\right\}}_{k,i,j=1}^{K,D_{in},D_{out}}=Conv\left[L,M\right]\left(\tilde{P},\Phi \right)\;\;\;\mathrm{with}\;\;g_{kij}=\sum_{l,m=1}^{L,M}{\tilde{p}}_{kml}\phi_{klmij}, \end{array}$$

where $\Phi =\{\phi_{klmij}\}$ is the parameter to learn. $Conv\left[L,M\right]\left(\tilde{P},\Phi \right)$ means convolution with kernel width, height as L,M, respectively, and the input of this convolution is $\tilde{P}$, Φ is the convolution kernels to learn. Note that $g_{kij}$ is polynomial, and after the network training, we will approximate the ideally effective convolution filters with learned parameters Φ guided by downstream tasks and loss function. Based on the learned convolution kernels *G*, we finally conduct convolution on the input point feature *F* and get the output of PSConv $\hat{F}\in R^{D_{out}}$ defined as

(4) $$\begin{array}{c} \hat{F}={\left\{\hat{f_{j}}\right\}}_{j=1}^{D_{out}}=Conv\left[K,D_{in}\right]\left(F,G\right),\;\;\;\mathrm{with}\;\;{\hat{f}}_{j}=\sum_{k,i=1}^{K,D_{in}}f_{ki}g_{kij}. \end{array}$$

Our PSConv is a novel convolution on a 3D point cloud with learned filters *G* based on linear transform followed by polynomial non-linearity over local points. Compared with the traditional convolutions that take non-linear transforms like ReLU and sigmoid to generate convolution filters in a limited range of values, our polynomial-based formulation can flexibly learn convolution filters with an unrestrictive range of values, because polynomials can theoretically approximate any smooth function [34]. As a layer defined on a point cloud, PSConv can be utilized for feature learning and inserted into any network for 3D point cloud analysis tasks.

### 3.2. Separable Formulation of PSConv

The bottleneck of directly conducting our convolution on a point cloud as in Equation (4) is the parameter size and computational cost. In this subsection, we propose an efficient strategy, i.e., a separable formulation of PSConv, to reduce the parameter size and computational complexity. A simple pipeline of our separable PSConv is presented in Figure 3, and now we introduce it in detail.

In Equations (3) and (4), the output feature dimension of PSConv is decided by Φ, and the full PSConv layer can be written as

(5) $$\begin{array}{c} {\hat{f}}_{j}=\sum_{k,i}^{K,D_{in}}f_{ki}\sum_{l,m=1}^{L,M}{\tilde{p}}_{klm}\phi_{klmij}, \end{array}$$

where $\Phi =\left\{\phi_{klmij}\right\}\in R^{K\times L\times M\times D_{in}\times D_{out}}$ is a five-dimensional weight matrix to learn, which also brings about huge computational costs to conduct. To reduce the parameter size of our point convolution, inspired by the separable convolution (e.g., FFT), we constrain that the element of this weight matrix can be decomposed to multiplication of elements from another two matrices $\tilde{\Phi }=\left\{{\tilde{\phi }}_{kij}\right\}\in R^{K\times D_{in}\times D_{out}}$ and $\hat{\Phi }=\left\{{\hat{\phi }}_{lmi}\right\}\in R^{L\times M\times D_{in}}$, with the original element of Φ separated by

$$\begin{array}{c} \phi_{klmij}={\hat{\phi }}_{lmi}{\tilde{\phi }}_{kij}. \end{array}$$

Note that with this separation, the parameter size is significantly reduced. By this assumption, we can rewrite Equation (5) as

(6) $$\begin{array}{cc} {\hat{f}}_{j} & =\sum_{k,i=1}^{K,D_{in}}f_{ki}\sum_{l,m=1}^{L,M}{\tilde{p}}_{klm}{\hat{\phi }}_{lmi}{\tilde{\phi }}_{kij}=\sum_{k,i=1}^{K,D_{in}}f_{ki}h_{ki}{\tilde{\phi }}_{kij}, \end{array}$$

where we represent $h_{ki}=\sum_{l,m=1}^{L,M}{\tilde{p}}_{kml}{\hat{\phi }}_{lmi}$.

Based on the separable formulation of Equation (6), we further introduce non-linear transform on $h_{ki}$ and define our separable formulation of PSConv as

(7) $$\begin{array}{cc} {\hat{f}}_{j} & =\sum_{k,i=1}^{K,D_{in}}f_{ki}\beta \left(h_{ki}\right){\tilde{\phi }}_{kij}=\sum_{k,i=1}^{K,D_{in}}{\hat{h}}_{ki}{\tilde{\phi }}_{kij}, \end{array}$$

where ${\hat{h}}_{ki}$ is the Hadamard product of $f_{ki}$ and $\beta (h_{ki})$, i.e., ${\hat{h}}_{ki}=f_{ki}\beta \left(h_{ki}\right)$. β is a non-linear transform composed of Batch Normalization (BN) and ReLU operations.

To present the progress clearly, in Figure 3, we illustrate the pipeline of our separable PSConv layer. Compared with the full convolution defined in Section 3.1, with our separable PSConv, we only need to learn parameters $\tilde{\Phi }=\left\{{\tilde{\phi }}_{kij}\right\}\in R^{K\times D_{in}\times D_{out}}$ and $\hat{\Phi }=\left\{{\hat{\phi }}_{lmi}\right\}\in R^{L\times M\times D_{in}}$ with less parameters. The computational complexity of separable PSConv with Equation (7) is $O(KLMD_{in})$, which is efficient to conduct with significantly less computational cost compared with the original PSConv in Equation (5) with computational complexity as $O(KLMD_{in}D_{out})$, and our separable PSConv is efficient to conduct. The separable PSConv can be taken as a basic layer to construct a network, and we take it as a basic layer to design our hierarchical PSNet.

### 3.3. PSNet

With PSConv as the basic layer, we introduce how to use it as a basic element to build our hierarchical PSNet for 3D point cloud analysis in this subsection. In Figure 2b, we present a pipeline of our PSNet, which consists of several stages and sampling operations as well as MLP and max-pooling. Specifically, in every stage, we first update pointwise features with the help of local KNN points, i.e., we take MLP and max-pooling operations within local KNN points to extract pointwise descriptors. Note that the MLP and max-pooling are basic operations in PointNet++ [5]. With the updated feature, we then apply four consecutive PSConv layers to strengthen the point descriptors, and their output features are concatenated as output for this stage.

With the basic stage described above, we construct our hierarchical PSNet as present in Figure 2b. Given the 3D shape, we first apply one basic stage (Stage 1) to extract point features, and then coarsen the shape by the Farthest Point Sampling with the same point sampling rate of 25%, followed by another basic stage (Stage 2) with the same structure as Stage 1 to update point features. More stages and sampling operations can be added to form a hierarchical architecture. In our PSNet, we use two stages.

Our PSNet can be applied to 3D point analysis tasks such as classification and segmentation. For shape classification, after the last stage, we further employ one shared MLP to all the point features and then max-pool them to form a shape descriptor, which is finally fed to the last MLP followed with a Softmax operation for shape category prediction. For shape segmentation, after the last stage, we further employ one shared MLP to all the point features and then max-pool them to form a global shape descriptor, which is then propagated from sparse points to dense points gradually based on distances within points. This feature propagation (FP) is also a basic component of PointNet++ [5], which consists of feature interpolation (FI) and MLP operations. We finally predict point labels with MLP and Softmax operation. Cross-entropy loss is applied to our PSNet for both shape classification and segmentation tasks. In Table A1 of the Appendix A, we list the details of our network such as the parameter size and architecture in every stage.

For PSNet, we take 1024 points for both shape classification and segmentation tasks. For PSConv, we set the parameters as L=10,M=3,K=16. We add ReLU after the linear transform of Equation (1), and we use BN and ReLU on the output of PSConv. When training our PSNet, the Adam optimizer is utilized, with the initial learning rate, epoch number, and batch size as 0.001, 250, and 32, respectively. The learning rate is exponentially decayed with a decay rate of 0.7 and decay step of 200,000. We utilize the data augmentation strategy as in [6] to train our network. That is, for point cloud classification, the point cloud is randomly rotated along the up-axis, and the position of each point is jittered by Gaussian noise with zero mean value and 0.01 standard deviation. While for segmentation, we only add the jittered noise. Clean data are utilized for the test of both classification and segmentation tasks.

## 4. Results

In this section, we first present the datasets and evaluation methods in Section 4.1 and then simply introduce the compared methods in Section 4.2. The experiment results are shown and discussed in Section 4.3, Section 4.4, Section 4.5, with a further discussion in Section 4.6. We also present ablation studies in Section 4.7 to show the effect of our design.

### 4.1. Datasets and Evaluation Methods

**Figure 4:**
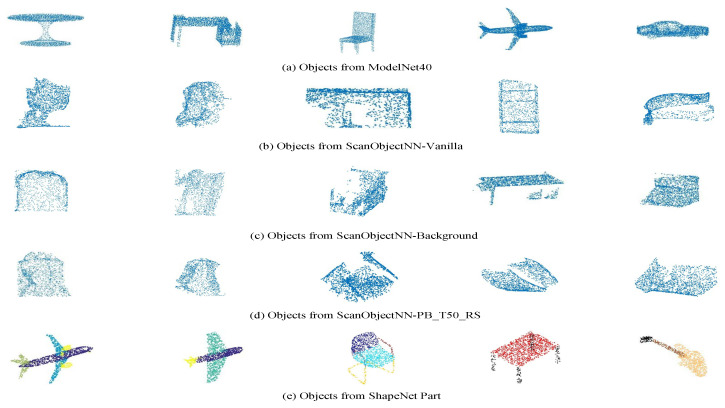
Shapes from ModelNet40, ScanObjectNN, and ShapeNet Part datasets. The categories are highly imbalanced for ModelNet40 and ShapeNet Part datasets, which are shown in the subfigures (**a**) and (**e**) respectively. The shapes in ScanObjectNN datasets are noisy and have geometric distortions, and we show shapes from the three variants of the ScanObjectNN dataset in (**b**–**d**). In this figure, for every dataset, the first two shapes are of the same category, and they have divergent appearances or point labels.

We apply our model to two fundamental 3D point cloud analysis tasks: shape classification and segmentation. For shape classification, we conduct experiments on the synthetic ModelNet40 [9] dataset and the scanned real-object ScanObjectNN [8] dataset. We also evaluate our model on Shapenet Part [22] dataset for the shape segmentation task. We list below the details and experiment setting for each dataset:

ModelNet40 [9]. It contains 12,311 CAD objects from 40 categories. We use the official split with 9843 shapes utilized for training and 2468 shapes for the test. We present several objects from this dataset in Figure 4a.

ScanObjectNN [8]. There are 2902 scanned real-world 3D objects in this dataset categorized into 15 classes. We use the standard split in our experiment, i.e., 80% and 20% of the data are utilized for training and testing, respectively. We utilize three variants, i.e., the ScanObjectNN-Vanilla, ScanObjectNN-Background, and ScanObjectNN-PB_T50_RS, to evaluate our method. The Vanilla and Background variants contain ground truth object and object with background points, respectively. The ScanObjectNN-PB_T50_RS contains an object with translation that randomly shifts up to 50% of its size as well as rotation and scaling transforms. Sample objects of these variants are shown in Figure 4b–d, respectively. The results of this dataset are from its official website.

ShapeNet Part [22]. This dataset contains 14,006/2874 training/test synthetic shapes from 16 categories of objects, with each point annotated with a label from 50 parts in total. We present several objects from this dataset in Figure 4e, where points with different colors represent points with different part labels.

For the synthetic ModelNet40 and ShapeNet Part datasets, the categories are highly imbalanced, which poses a challenge to all methods including ours, and different shapes of the same category (e.g., first two columns in Figure 4a) may have significantly different appearances. Different shapes (e.g., first two columns in Figure 4e) may have divergent numbers of part labels. For the real scanned ScanObjectNN datasets, point clouds are noisy, as shown in Figure 4b–d, and the objects have geometric distortions, such as holes, which are extremely challenging to recognize.

Evaluation Methods. For shape classification, the results are evaluated by Overall Accuracy (OA) and mean per-class accuracy (mACC), i.e., the percentage of correctly classified shapes over all shapes and the mean classification accuracy over all categories. For the shape segmentation task, we report the Intersection-over-Union (IoU) accuracy averaged across all part classes, which measures the overlap between correct predictions and ground truth labels. These measures are widely utilized for 3D shape classification and segmentation tasks [4,5,7,9,14,22].

### 4.2. Compared Methods

In this subsection, we present the compared methods of our PSNet for shape analysis, which mainly focus on designing convolutions on 3D point clouds, including PointNet [4], PointNet++ [5], SpiderCNN [6], PointCNN [7], RSCNN [13], DGCNN [14], SPLATNet [16], KPConv [18], InterpConv [19], PointWeb [20], PointConv [21], PointGrid [23], A-CNN [24], 3DmFV [25], KD-Net [35]. As described in the Related Work section, for the methods of PointNet [4], PointNet++ [5], RSCNN [13], and DGCNN [14], they all first update local point features and then aggregate features by max-pooling operation. For the methods of PointCNN [7], SPLATNet [16], KPConv [18], InterpConv [19], PointGrid [23], A-CNN [24] and 3DmFV [25], they represent points with regular formations by reorder operation or discrete representation. For the methods of SpiderCNN [6], PointWeb [20] and PointConv [21], they design local convolution filters and then conduct convolution on the local points. In addition to the above methods, we also compare with KD-Net [35], which performs multiplicative transformations and shares parameters of these transformations according to the subdivisions of the point clouds imposed onto them by kd-trees. For shape classification on ScanObjectNN [8], we also compare with BAG-PN++ and BAG-DGCNN, which are methods in [8].

These methods are similar to our method, which is also based on the design of point convolution as well as hierarchical architecture. We compare them in order to demonstrate the effectiveness and novelty of our method. Among these methods, SpiderCNN [6], PointWeb [20] and PointConv [21] are the three most related works to ours, which first design convolution kernels and then conduct point convolution on a point cloud.

### 4.3. Shape Classification on ModelNet40

We first evaluate PSNet for shape classification on the ModelNet40 [9] dataset. We compare our PSNet with state-of-the-art methods and present the classification accuracies in Table 1. Our PSNet taking 1024 points as input achieves the best OA and mACC results among the compared methods, and it achieves better accuracies even compared with those methods using 5000/6800 points. These comparisons verify that our proposed network is effective for the point cloud classification task.

Note that the baseline of our network is PointNet++ [5], whose basic components are MLP, max-pooling and sampling operation, as in our PSNet. Compared with PointNet++, PSNet achieves 93.1% OA classification accuracy, with a significant 2.4% increased accuracy. This comparison demonstrates the effectiveness of our PSConv layer for local point feature extraction.

Furthermore, compared with these methods that design convolution kernels, including SpiderCNN [6], PointWeb [20] and PointConv [21], our method performs better with at least 0.6% higher OA accuracy, and this proves the efficacy of our polynomial-based strategy for the learning of convolution kernels. This higher accuracy can be explained by the effectiveness of the polynomials because they are more flexible and can approximate any smooth functions theoretically. When being utilized in local point convolution, they can capture the geometric information hidden behind the unstructured points.

### 4.4. Shape Classification on ScanObjectNN

We further apply our PSNet on the ScanObjectNN-Vanilla, ScanObjectNN-Background and ScanObjectNN-PB_T50_RS datasets and report classification results in Table 2. Compared with state-of-the-art methods, PSNet achieves the highest accuracies on all the datasets for both OA and mACC measures, which prove the efficacy of our PSNet for analysis of scanned objects in the real world.

Compared with the baseline method PointNet++ [5], our PSNet gains improvements with 2.3%, 4.3%, 4.3% higher OA and 2.2%, 3.5, 3.2% higher mACC accuracies, respectively, on these three datasets. These comparisons show that our PSConv layer is an effective layer to learn a point feature.

We also present per-class accuracies on the three variants of the ScanObjectNN dataset in Table A2, Table A3, Table A4 of the Appendix, respectively, where our PSNet also performs the best in many categories and outperforms the compared methods.

Considering that the ScanObjectNN dataset consists of scanned real-world objects with noise and geometric distortions, these results and comparisons all demonstrate the effectiveness of our method in real data analysis tasks and in real-world applications.

### 4.5. Shape Segmentation on ShapeNet Part

We finally apply PSNet for the 3D point cloud segmentation task on the ShapeNet Part [22] dataset to predict point labels. We present the IoU accuracies in Table 3. As shown in the table, our PSNet achieves better performance than most of the methods, and it also achieves competitive accuracy with KPConv [18], which takes about 2300 k points as input compared with ours taking 1024 points as input. We also present per-class IoU in this table, and PSNet performs the best on categories of chair, knife, and rocket, etc.

Compared with the baseline method PointNet++ [5], our PSNet achieves 1.1% higher mean IoU on this dataset, and this demonstrates the effectiveness of our PSConv layer for point feature extraction. Compared with the works that design point convolution kernels, such as SpiderCNN [6] and PointConv [21], our method based on polynomials presents better performance.

**Figure 5:**
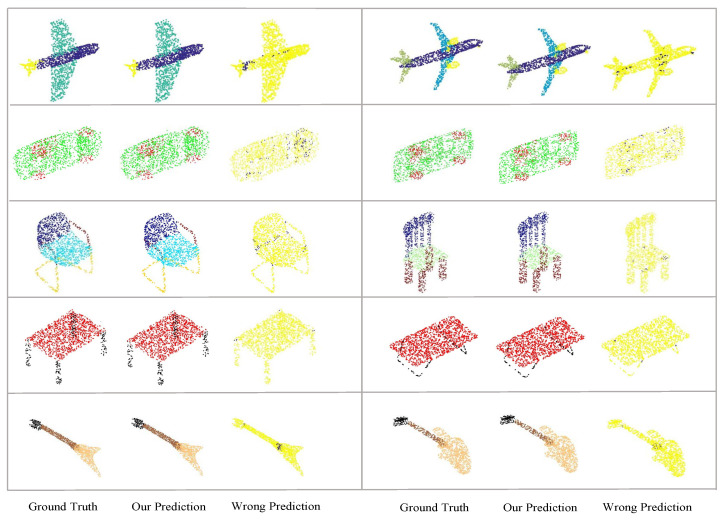
Shape segmentation results. In every box, we present the object with ground truth labels and our predicted labels in the first and second columns, respectively. We also highlight the wrongly predicted points with dark blue color, which are mainly located near the connection of two parts in one shape.

In Figure 5, we show the segmentation results of several objects in the ShapNet Part dataset as well as the corresponding ground truth labels for every object. We also show the predicted incorrect labels in the last column in every box, which are highlighted by a dark blue color. As illustrated in the figure, our predicted labels are reasonable and close to the ground truth, and the points with predicted incorrect labels are mainly near the connection of two parts, which are really hard and indistinct to predict.

### 4.6. Ablation Study

In this subsection, we conduct an ablation study on PSNet to justify the effects of our network design, including the effect of linear transform and power operation, the effect of polynomials in the PSConv layer, and the effect of layer number and stage number. We take the baseline PSNet with one stage consisting of four layers of PSConv, which achieves 92.8% OA accuracy on ModelNet40 [9].

Effect of linear transform and power operation. To prove the effect of linear transform and power operation in our PSConv layer, in Table 4, we present the results of our PSNet without linear transform and power operation in the PSConv layer, respectively, i.e., PSNet-noL-trans and PSNet-noPower, and their accuracies are 92.4% and 92.3%, respectively, which are lower than our full PSNet model with 92.8% accuracy. These comparisons show the necessities of our linear transform and power operation in the design of a PSConv layer.

Effect of polynomial. In the design of our PSConv layer, the convolution filters are learned based on polynomial as in Equation (1)–(3), and now we present the results of PSNet with a polynomial replaced by other operations to prove its effect. We replace our polynomial with operations such as Linear transform (L-trans.), ReLU, Sigmoid (Sig.), Tanh, Leaky-ReLU (L-ReLU), Exp, FC (followed by BN and ReLU) [36], which are followed by a traditional convolution to learn filters as in Equation (3). We present the results in Table 5, and our method achieves the best performance among the compared methods, showing that our strategy with a polynomial is more effective than that with traditional non-linear operations.

**Figure 6:**
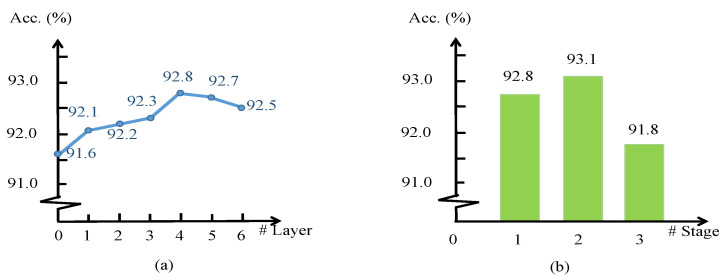
Classification accuracies on ModelNet40 (OA in %) with (**a**) different amounts of PSConv layers in one stage and (**b**) different amounts of stages in PSNet. As shown in the figure, PSNet with 4 layers of PSConv and 2 stages achieves the highest accuracies.

Effect of PSConv layer number and stage number. In our PSNet, we take four sequential PSConv layers in one stage and two stages in PSNet. To show the effect of PSConv layer number and stage number, we present classification results of PSNet in Figure 6a with different layer numbers in one stage and (b) with different stage numbers in PSNet. As demonstrated in the figure, PSNet with four layers of PSConv and two stages achieves the highest accuracies. More layers and more stages will not get higher accuracies in the design of our PSNet.

**Figure 7:**
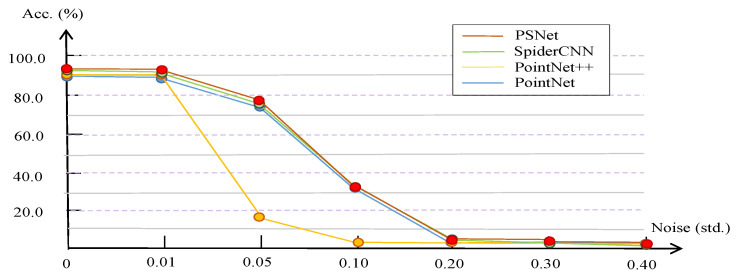
Ablation study for robustness of PSNet with different levels of noise. Our PSNet remains robust with a noise level of 0.01. Compared with SpiderCNN, PointNet++ and PointNet, our PSNet achieves better performances under noise levels of 0.01, 0.05, 0.10 and 0.30.

Robustness to noise. To justify the robustness of our PSNet, we train PSNet on the ModelNet40 training dataset and test it on the test data of ModelNet40 with various levels of noise. We add Gaussian noise with a mean value of zero and with a different standard deviation (Std) on each point (coordinates within a unit ball) independently. The OA accuracies are presented in Figure 7. As shown in the figure, our PSNet keeps robustness under noise with a Std of 0.01. In this figure, we also present the accuracy lines of PointNet [4], PointNet++ [5] and SpiderCNN [6] for comparisons. The performances of all of these methods, including ours, drop with the increase in noise level. Compared with the other methods, our PSNet performs better with noise levels of 0.01, 0.05, 0.10 and 0.30.

### 4.7. Discussion

In this subsection, we systematically compare our method with the related point cloud networks in methodology to analyze the distinctive characteristics, novelties and explanation of the effectiveness of our approach.

We first compare our method with the baseline method PointNet++ [5], whose basic components are MLP, max-pooling and sampling operation, as in our network. Our PSNet differs from PointNet++ with the additional PSConv layers. As shown in Section 4.3, Section 4.4, Section 4.5, PSNet achieves significantly higher accuracies for both shape classification and segmentation tasks on various standard datasets. These increased accuracies are mainly attributed to our PSConv layer. This comparison indicates that the PSConv layer is an effective operation for point feature extraction, which helps for shape analysis tasks.

We then compare our method with point-based methods that design various point convolutions, including PointNet [4], PointNet++ [5], SpiderCNN [6], PointCNN [7], RSCNN [13], DGCNN [14], SPLATNet [16], KPConv [18], InterpConv [19], PointWeb [20], PointConv [21], PointGrid [23], A-CNN [24], 3DmFV [25], KD-Net [35], BAG-PN++ [8] and BAG-DGCNN [8]. These methods commonly first design point convolution for feature extraction and then construct hierarchical network architectures for the 3D shape analysis tasks. For our PSNet, we also design a hierarchical network, i.e., PSNet, for 3D point cloud classification and segmentation. However, our method differs from them with an innovative point convolution, i.e., PSConv layer. Compared with these methods, our PSNet with PSConv layer can better capture local shape information because we designed it with flexible convolution kernels trained on polynomials. Our design benefits from the approximation ability of polynomials. As shown in Section 4.3, Section 4.4, Section 4.5, compared with these point-based methods, our PSNet achieves the best classification performance and competitive segmentation performance.

We next compare our separable PSConv with the full formulation of PSConv and the previous separable convolution. For the full formulation of PSConv, we need to learn parameter $\Phi \in R^{K\times L\times M\times D_{in}\times D_{out}}$. While for our separable PSConv, we only need to learn $\tilde{\Phi }=\left\{{\tilde{\phi }}_{kij}\right\}\in R^{K\times D_{in}\times D_{out}}$ and $\hat{\Phi }=\left\{{\hat{\phi }}_{lmi}\right\}\in R^{L\times M\times D_{in}}$ with significantly less parameters. For the computational complexity, it is $O(KLMD_{in}D_{out})$ for the full PSConv, while it is reduced to $O(KLMD_{in})$ for our separable PSConv. With our separable PSConv layer, it is efficient to conduct 3D shape analysis with remarkably lower parameter size and computational complexity. For our separable PSConv, we would like to highlight that our separable formulation is different from the previous spatial separable convolutions such as FFT and [29] as well as the depthwise separable convolution [30,31,32]. We do not explicitly split the convolution spatially or split it into depthwise and pointwise ones but advance it by separating the convolution kernels into a flexible and adaptive combination. We design our novel separable formulation flexibly to construct the point convolution, which may offer a new strategy for the design of separable convolution.

We finally compare our polynomial-based transform with traditional transforms for the convolution kernel learning of our PSConv layer. Compared with the traditional transforms [36] such as Linear transform, ReLU, Sigmoid, Tanh, Leaky-ReLU, Exp, and FC, etc., the polynomials can theoretically approximate any smooth function with an unrestrictive range of values. With polynomial-based transform, our PSConv layer can better explore the local geometric information hidden behind the irregular local points. The advantage of our polynomial-based strategy for convolution kernel learning is also proved by the results in Section 4.6, e.g., PSConv based on polynomials achieves at least 0.26% higher OA classification accuracy than PSconv based on the other transforms.

In summary, our approach is well motivated by the polynomial approximation of convolution kernels, and it can well reduce the network parameter size as well as computational complexity by separable formulation. Compared with the previous convolutions, our approach based on the above innovations has achieved advantageous performance for shape classification and competitive accuracy for shape segmentation.

## 5. Conclusions

With the development of 3D sensors, shape classification and segmentation are two major tasks for the application of 3D point clouds. Designing an effective and efficient point convolution is necessary for feature extraction, which is the target of our work.

In this paper, we first design a novel point convolution, i.e., PSConv, on a 3D point cloud. It is designed based on polynomials of transformed local point coordinates. The polynomial-based kernels with learned parameters are able to approximate ideal convolution kernels with the guidance of loss function by network training. Compared with previous methods, our polynomial-based strategy can better capture the local geometric shape information. To reduce the parameter size and computational cost, we further construct a separable formulation of the PSConv layer. The separable PSConv can be efficiently applied while retaining efficacy, making it capable of building a multi-layer deep convolutional network on 3D point clouds. With PSConv as a basic layer, we design the hierarchical PSNet for point analysis. We evaluate it on standard synthetic and real scanned datasets, and it achieves state-of-the-art results for shape classification. It also has competitive performance for point cloud segmentation tasks.

However, there are limitations to our method that need further exploration. Firstly, with the reduction of parameter size in our separable formulation of PSConv, the representation ability may be reduced, which inspires us to design it with more flexibility and capability in future work. Secondly, although PSNet has achieved the highest accuracy in the real scanned ScanObjectNN dataset, with the increase of noise level, the performance drops. This phenomenon is also observed in the experiment on the ModelNet40 dataset, and this is also the challenge for all the point-based methods. To overcome this difficulty, a more stable and effective strategy for point convolution should be designed. Thirdly, PSConv is designed to operate on a local point cloud, and we design PSNet for 3D object analysis. However, for the applications in indoor and outdoor scenes, a more effective network architecture on large-scale point clouds is essential.

For our future work, it is worthwhile thinking about how to deal with the limitations. To improve the capability for our separable PSConv layer, we plan to introduce the multi-head strategy when separating the parameters, which may better balance the trade-off between computation cost and representation ability. To improve the stability of our method, designing robust point convolution based on polynomials by explicitly handling the outliers is one of our future research directions. To apply the PSConv layer to large-scale point clouds, we would like to incorporate our PSConv into mainstream image convolution network architectures, such as ResNet [37] and DenseNet [38]. Furthermore, we are also interested in applying our PSNet for other applications, such as detection, completion and registration, etc., to explore the potential of our PSConv layer. 

## Figures and Tables

**Table 1:** Shape classification accuracies on ModelNet40 (OA and mACC in %). Our PSNet with 1024 points achieves the best accuracy even compared with the methods using 5000/6800 points.

Method	# Input	OA	mACC
PointNet [4]	1024	89.1	86.2
PointNet++ [5]	1024	90.7	–
PointCNN [7]	1024	92.5	88.1
RSCNN [13]	1024	91.7	–
DGCNN [14]	1024	92.2	90.2
InterpConv [19]	1024	93.0	–
PointWeb [20]	1024	92.3	89.4
PointConv [21]	1024	92.5	–
PointGrid [23]	1024	92.0	88.9
A-CNN [24]	1024	92.6	90.3
3DmFV [25]	1024	91.4	86.3
PointNet++ [5]	5000	91.9	–
SpiderCNN [6]	5000	92.4	86.8
KD-Net [35]	5000	91.8	88.5
KPConv-rigid [18]	6800	92.7	–
KPConv-deform [18]	6800	92.9	–
Proposed	1024	**93.1**	**90.4**

**Table 2:** Shape classification accuracy (OA and mACC in %) on ScanobjectNN-Vanilla (SV), ScanobjectNN-Background (SB), and ScanObjectNN-PB_T50_RS (SP). Our PSNet performs better than all of the compared methods for both OA and mACC measures on all the datasets.

Measure	Dataset	PointNet [4]	PointNet++ [5]	SpiderCNN [6]	PointCNN [7]	DGCNN [14]	3DmFV [25]	Ours
OA	SV	79.2	84.3	79.5	85.5	86.2	73.8	**86.6**
	SB	73.3	82.3	77.1	86.4	82.8	68.2	**86.6**
	SP	68.2	77.9	73.7	78.5	78.1	63.0	**82.2**
mACC	SV	74.4	82.1	77.4	83.3	84.0	68.9	**84.3**
	SB	69.4	79.9	72.4	83.3	78.8	58.8	**83.4**
	SP	63.4	75.4	69.8	75.1	73.6	58.1	**78.6**

**Table 3:** Shape segmentation IoU on the ShapeNet Part dataset (in %). Our PSNet achieves competitive accuracies, and it also performs best on the categories of chair, knife, rocket and table.

Method	Mean	Aero	Bag	Cap	Car	Chair	Earph.	Guitar	Knife	Lamp	Laptop	Motor	Mug	Pistol	Rocket	Skate	Table
PointNet [4]	83.7	83.4	78.7	82.5	74.9	89.6	73.0	91.5	85.9	80.8	95.3	65.2	93.0	81.2	57.9	72.8	80.6
PointNet++ [5]	85.1	82.4	79.0	87.7	77.3	90.8	71.8	91.0	85.9	83.7	95.3	71.6	94.1	81.3	58.7	76.4	82.6
SpiderCNN [6]	85.3	83.5	81.0	87.2	77.5	90.7	76.8	91.1	87.3	83.3	95.8	70.2	93.5	82.7	59.7	75.8	82.8
PointCNN [7]	86.1	84.1	**86.5**	86.0	80.8	90.6	79.7	92.3	88.4	85.3	96.1	77.2	95.3	84.2	64.2	80.0	83.0
RSCNN [13]	86.2	83.5	84.8	**88.8**	79.6	91.2	**81.1**	91.6	88.4	**86.0**	96.0	73.7	94.1	83.4	60.5	77.7	83.6
DGCNN [14]	85.1	84.2	83.7	84.4	77.1	90.9	78.5	91.5	87.3	82.9	96.0	67.8	93.3	82.6	59.7	75.5	82.0
SPLATNet3D [16]	84.6	81.9	83.9	88.6	79.5	90.1	73.5	91.3	84.7	84.5	**96.3**	69.7	95.0	81.7	59.2	70.4	81.3
SPLATNet2D-3D [16]	85.4	83.2	84.3	89.1	80.3	90.7	75.5	92.1	87.1	83.9	96.3	75.6	95.8	83.8	64.0	75.5	81.8
KPConv-rigid [18]	86.2	83.8	86.1	88.2	**81.6**	91.0	80.1	92.1	87.8	82.2	96.2	77.9	95.7	**86.8**	65.3	81.7	83.6
KPConv-deform [18]	**86.4**	**84.6**	86.3	87.2	81.1	91.1	77.8	**92.6**	88.4	82.7	96.2	**78.1**	**95.8**	85.4	**69.0**	**82.0**	83.6
PointConv [21]	85.7	–	–	–	–	–	–	–	–	–	–	–	–	–	–	–	–
KD-Net [35]	82.3	80.1	74.6	74.3	70.3	88.6	73.5	90.2	87.2	81.0	94.9	57.4	86.7	78.1	51.8	69.9	80.3
Proposed	86.2	83.5	85.4	86.5	79.8	**91.3**	78.0	91.4	**88.6**	84.5	96.1	72.2	95.0	83.6	**69.0**	75.7	**83.8**

**Table 4:** Ablation study on linear transform and power operation in the PSConv layer. We present shape classification accuracy on ModelNet40 (OA in %). L-Trans. denotes linear transform. The model with both linear transform and power operation performs best.

Method	L-Trans.	Power	Acc.
PSNet-noL-trans	×	√	92.4
PSNet-noPower	√	×	92.3
PSNet	√	√	92.8

**Table 5:** Ablation study on polynomial in PSConv layer. We report shape classification accuracy on ModelNet40 (OA in %). Sig. denotes Sigmoid operation, L-trans. is short for linear transform. Our design with polynomials achieves the best accuracy.

L-Trans.	ReLU	Sig.	Tanh	L-ReLU	Exp	FC	Ours
92.32	92.45	92.51	92.47	92.53	92.34	92.34	**92.79**

## Data Availability

Data sharing not applicable.

## Appendix A

### Appendix A.1

We present the details of our network for shape classification and segmentation tasks in Table A1, such as the parameter size and architecture in every stage. Note that feature propagation is only utilized for the shape segmentation task.

**Table A1:** Network details of PSNet. Numbers in each bracket represent the numbers of hidden units in successive hidden layers. For the PSConv layer, we list the output channel dimensions. FP denotes Feature Propagation and FI denotes Feature Interpolation, which are operations for point segmentation. MLP* denotes MLP operation after Stage 2, and MLP** is the last MLP for label prediction.

	Architecture	Parameters Size	
		Classification	Segmentation
Stage 1	MLP →Max-pooling → PSConv1 → PSConv2 → PSConv3 → PSConv4 →	MLP:(32,32,64) PSConv1:(64) PSConv2:(64) PSConv3:(64) PSConv4:(64)	MLP:(32,32,64) PSConv1:(64) PSConv2:(64) PSConv3:(64) PSConv4:(64)
	Concatenate		
Stage 2	MLP → Max-pooling → PSConv1 → PSConv2 → PSConv3 → PSConv4 →	MLP:(64, 64, 128) PSConv1:(128) PSConv2:(128) PSConv3:(128) PSConv4:(128)	MLP:(64, 64, 128) PSConv1:(128) PSConv2:(128) PSConv3:(128) PSConv4:(128)
	Concatenate		
MLP*	MLP	MLP:(256, 512, 1024)	MLP:(256, 512, 1024)
FP	FI1 → MLP1 → FI2 → MLP2 → FI3 → MLP3	N/A	MLP1:(256, 256) MLP2:(256,128) MLP3:(128,128)
MLP**	MLP	MLP:(512, 256, 40)	MLP:(512, 256, 128, 50)

### Appendix A.2

We present the per-class classification results of ScanObjectNN-Vanilla, ScanObjectNN-Background and ScanObjectNN-PB_T50_RS datasets in Table A2, Table A3, Table A4, respectively, where our PSNet also performs the best in many categories. Our PSNet performs best on 7/9/10 sub-categories, and it outperforms the second best work PointCNN with the highest score on 6/7/2 sub-categories. Furthermore, the mean accuracies across all sub-categories of PSNet is 1.0%/0.1%/3.5% higher than PointCNN. Compared with other methods, our method also has better performance.

**Table A2:** Per-class accuracies (in %) on the ScanobjectNN-Vanilla dataset. PSNet achieves the highest mACC accuracy. It also performs better than all of the compared methods for the categories of box, cabinet, chair, and sink, etc.

Method	mACC	Bag	Bin	Box	Cabinet	Chair	Desk	Display	Door	Shelf	Table	Bed	Pillow	Sink	Sofa	Toilet
PointNet [4]	74.4	47.1	80.0	35.7	80.0	93.6	**86.7**	73.8	**97.6**	81.6	**88.9**	59.1	76.2	54.2	85.7	76.5
PointNet++ [5]	82.1	**70.6**	**90.0**	35.7	84.0	96.2	83.3	81.0	88.1	83.7	87.0	86.4	85.7	79.2	92.9	88.2
SpiderCNN [6]	77.4	52.9	80.0	35.7	64.0	96.2	73.3	83.3	**97.6**	81.6	83.3	77.3	**90.5**	75.0	88.1	82.4
PointCNN [7]	83.3	64.7	**90.0**	46.4	82.7	98.7	83.3	**85.7**	88.1	**85.7**	**88.9**	86.4	**90.5**	70.8	92.9	**94.1**
DGCNN [14]	84.0	76.5	**90.0**	64.3	80.0	98.7	80.0	**85.7**	88.1	91.8	87.0	**90.9**	**90.5**	70.8	**95.2**	70.6
3DmFV [25]	68.9	41.2	82.5	32.1	66.7	98.7	53.3	71.4	90.5	73.5	75.9	59.1	81.0	58.3	90.5	58.8
Proposed	**84.3**	52.9	85.0	**75.0**	**85.3**	**100.0**	80.0	**85.7**	95.2	77.6	**88.9**	86.4	85.7	**79.2**	**95.2**	91.7

**Table A3:** Per-class accuracies (in %) for ScanobjectNN-Background. PSNet achieves the highest mACC accuracy. It also performs better than all of the compared methods for the categories of box, cabinet, desk, display, shelf, and toilet, etc.

Method	mACC	Bag	Bin	Box	Cabinet	Chair	Desk	Display	Door	Shelf	Table	Bed	Pillow	Sink	Sofa	Toilet
PointNet [4]	69.4	47.1	65.0	25.0	65.3	93.6	50.0	76.2	**95.2**	87.8	72.2	77.3	76.2	62.5	83.3	64.7
PointNet++ [5]	79.9	52.9	90.0	39.3	80.0	96.2	80.0	78.6	90.5	83.7	74.1	**90.9**	85.7	**87.5**	92.9	76.5
SpiderCNN [6]	72.4	41.2	80.0	25.0	82.7	97.4	56.7	78.6	92.9	73.5	75.9	81.8	81.0	70.8	83.3	64.7
PointCNN [7]	83.3	**58.8**	**95.0**	50.0	82.7	**100.0**	73.3	83.3	90.5	89.8	**83.3**	**90.9**	**90.5**	79.2	**100.0**	82.4
DGCNN [14]	78.8	52.9	90.0	50.0	85.3	96.2	73.3	85.7	92.9	85.7	81.5	72.7	81.0	79.2	85.7	70.6
3DmFV [25]	61.6	58.8	65.0	17.9	69.3	96.2	23.3	83.3	88.1	65.3	72.2	45.5	52.4	54.2	85.7	47.1
Proposed	**83.4**	35.3	87.5	**71.4**	**88.0**	94.9	**83.3**	**88.1**	**95.2**	**91.8**	**83.3**	86.4	**90.5**	79.2	92.9	**83.3**

**Table A4:** Per-class accuracies (in %) for ScanobjectNN-PB_T50_RS. PSNet achieves the best mACC accuracy. It also performs better than all of the compared methods for the categories of box, desk, display, door, shelf, table, bed, pillow, and sofa, etc.

Method	mACC	Bag	Bin	Box	Cabinet	Chair	Desk	Display	Door	Shelf	Table	Bed	Pillow	Sink	Sofa	Toilet
PointNet [4]	63.4	36.1	69.8	10.5	62.6	89.0	50.0	73.0	93.8	72.6	67.8	61.8	67.6	64.2	76.7	55.3
PointNet++ [5]	75.4	49.4	84.4	31.6	77.4	91.3	74.0	79.4	85.2	72.6	72.6	75.5	81.0	**80.8**	90.5	**85.9**
SpiderCNN [6]	69.8	43.4	75.9	12.8	74.2	89.0	65.3	74.5	91.4	78.0	65.9	69.1	80.0	65.8	90.5	70.6
PointCNN [7]	75.1	**57.8**	82.9	33.1	83.6	92.6	65.3	78.4	84.8	84.2	67.4	80.0	80.0	72.5	91.9	**85.9**
BAG-PN++ [8]	77.5	54.2	**85.9**	39.8	81.7	90.8	76.0	84.3	87.6	78.4	74.4	73.6	80.0	77.5	91.9	**85.9**
BAG-DGCNN [8]	75.7	48.2	81.9	30.1	**84.4**	92.6	77.3	80.4	92.4	80.5	74.1	72.7	78.1	79.2	91.0	72.9
DGCNN [14]	73.6	49.4	82.4	33.1	83.9	91.8	63.3	77.0	89.0	79.3	77.4	64.5	77.1	75.0	91.4	69.4
3DmFV [25]	58.1	39.8	62.8	15.0	65.1	84.4	36.0	62.3	85.2	60.6	66.7	51.8	61.9	46.7	72.4	61.2
Proposed	**78.6**	25.3	69.8	**63.2**	79.0	**95.1**	**84.7**	**87.7**	**95.7**	**85.5**	**77.8**	**91.8**	**86.7**	77.5	**93.3**	66.3
